# Youth anxiety in an age of uncertainty: future insecurity, career stress, and the mechanisms of identity exploration during major life transitions

**DOI:** 10.3389/fpsyt.2026.1741101

**Published:** 2026-04-15

**Authors:** Yutong Sun

**Affiliations:** School of Educational Sciences, Nanjing Normal University, Nanjing, China

**Keywords:** career stress, emerging adulthood, future anxiety, identity exploration, rural normative pressure, social comparison, well-being

## Abstract

This study examines how young adults in China experience well-being under conditions of social uncertainty, focusing on the joint associations among future anxiety, career stress, identity exploration, rural normative pressure, and subjective well-being. Informed by cultural-psychological perspectives, the proposed model integrates multiple indirect associations and contextual moderation within a single structural framework. Using survey data from 260 undergraduate students, structural equation modeling and regression-based analyses showed that future anxiety was positively associated with career stress (β = 0.637, p <.001) and identity exploration (β = 0.346, p <.001), and negatively associated with well-being (β = -0.383, p <.001). career stress was positively associated with identity exploration (β = 0.316, p <.001) and negatively associated with well-being (β = -0.300, p <.001), whereas identity exploration was positively associated with well-being in the multivariate model (β = 0.265, p <.001). Bootstrap analyses further indicated a significant negative indirect association between future anxiety and well-being through career stress (β = -0.190, p <.001), a significant positive indirect association through identity exploration (β = 0.091, p <.001), and a significant sequential indirect association through career stress and identity exploration (β = 0.053, p <.001), although the total indirect effect was not significant. Rural normative pressure significantly but modestly moderated the association between career stress and identity exploration (β = 0.105, p = .040), such that this positive relationship became stronger at higher levels of normative pressure. Overall, the findings suggest that future-oriented uncertainty, career-related pressure, and identity-related reflection are closely intertwined in young people’s lives, and that sociocultural norms shape how pressure is translated into developmental self-reflection. The results underscore the importance of educational and social interventions that support not only stress reduction, but also identity-related meaning-making and context-sensitive developmental adaptation in uncertain social environments.

## 1 Introduction

Over the past decade, societies across the globe have undergone a series of profound and enduring structural transformations, including intensified economic volatility, rapid technological change, and the widespread reconfiguration of labor and social institutions. Across different national and regional contexts, these shifts have reshaped young people’s pathways into adulthood in distinct ways. In many Western societies, the combined effects of financial crises, inflationary pressures, and labor-market flexibilization have increased the prevalence of temporary employment and career instability among youth, reflecting a broader erosion of stable work experiences in contemporary economies (1). In East Asian contexts, prolonged educational competition and persistently high housing costs have significantly delayed transitions to economic independence and family formation. In parts of the Global South, structural unemployment and limited social protection have exposed young people to sustained livelihood uncertainty during the education-to-work transition. More broadly, global evidence suggests that economic uncertainty is closely linked to deteriorating mental health outcomes across societies, indicating that structural instability has become an important contextual factor shaping psychological well-being (2). Despite substantial institutional and cultural variation, a common pattern emerges: for young people worldwide, the predictability of future life trajectories has declined, and uncertainty has increasingly become a normalized social condition rather than a temporary risk. Cross-national research further indicates that mental health outcomes vary systematically across demographic and social contexts, highlighting how psychological well-being is embedded within broader structural conditions (3). In addition, rapid technological transformation and shifting labor-market expectations have made career preparation itself increasingly uncertain, complicating young people’s efforts to anticipate future occupational pathways (4). Research on future time perspective further shows that how individuals perceive and evaluate the future plays an important role in shaping career decision-making processes (5).

The COVID-19 pandemic further amplified this global context of uncertainty. As a transnational public crisis, the pandemic not only disrupted educational systems, labor markets, and channels of social mobility in the short term, but also reinforced young people’s perceptions of constrained opportunities and diminished personal control over the future. Empirical evidence from university populations indicates that anxiety and depression increased substantially during the pandemic, reflecting the psychological impact of prolonged uncertainty and disrupted life planning (6). Longitudinal research has also shown that the pandemic was associated with significant changes in adolescent mental health, with both risk and protective factors influencing psychological outcomes over time (7). Within this broader setting, youth anxiety has increasingly extended beyond an individual emotional response and taken on characteristics closely tied to social structures, institutional arrangements, and generational positioning. From a developmental psychological perspective, such structural uncertainty closely coincides with the life stage young people occupy. Emerging adulthood is characterized by identity exploration, career decision-making, and future planning—developmental tasks that inherently involve ambiguity and psychological tension (8). When social environments fail to provide stable institutional reference points or clear life-course expectations, young people are more likely to experience sustained anxiety while navigating these tasks. Rather than reflecting a temporary emotional disturbance, anxiety in this context becomes embedded in ongoing processes of self-definition, future orientation, and psychological adaptation. Recent research further indicates that intolerance of uncertainty and resilience processes are closely associated with future anxiety and mental well-being among young adults facing unstable futures (9).

**Figure 1:**
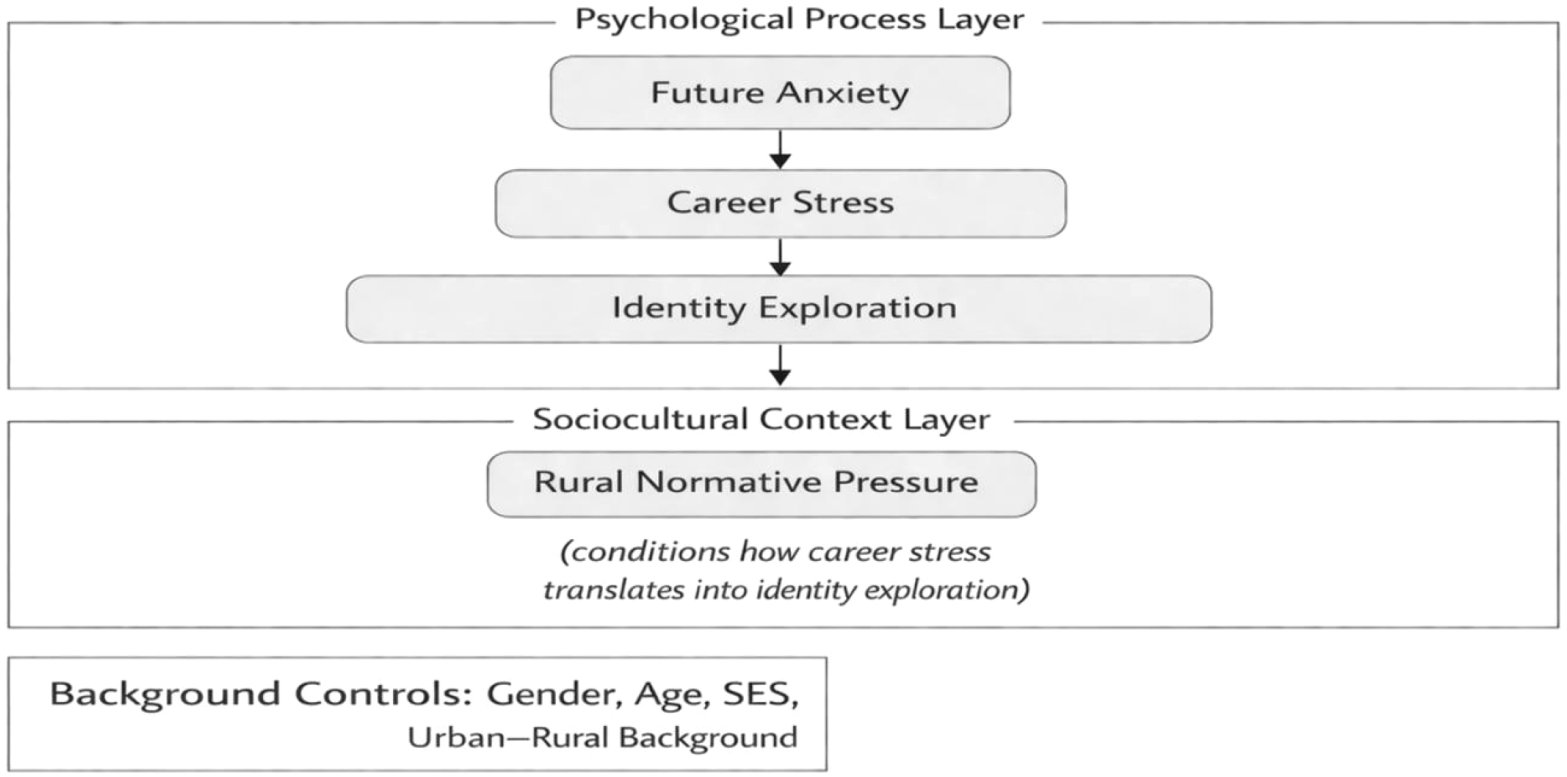
Overview of key constructs and analytical framework.

Despite growing scholarly attention to youth anxiety in relation to pandemics, economic risk, and developmental transitions, existing research exhibits several notable limitations. First, uncertainty is often treated as a singular external stressor, with insufficient attention to how structural conditions interact with individual psychological processes over time. Second, much of the literature focuses on describing anxiety levels or adverse mental health outcomes, while paying less attention to how young people actively negotiate uncertainty through developmental mechanisms such as identity exploration and career-related coping. Some studies have examined relationships between future time perspective, career adaptability, and career anxiety among university students, suggesting that career-related psychological resources may influence how uncertainty is experienced (10). At the same time, recent integrative reviews of identity development research highlight the growing complexity of identity processes in emerging adulthood, yet these perspectives have rarely been integrated into broader models of youth anxiety and future uncertainty (11). Longitudinal evidence also suggests that identity processes are closely associated with mental health outcomes in young adulthood, particularly through mechanisms such as social support and psychological adjustment (12). Third, contextual normative forces particularly those rooted in rural or traditional social environments remain underexamined, even though they may significantly shape how young people interpret stress, make life decisions, and evaluate personal success. In response to these gaps, the present study adopts a combined social and developmental perspective to examine how future-oriented uncertainty shapes youth psychological adaptation. Specifically, the study investigates the associations among career stress, identity exploration, and well-being, while also testing whether rural normative pressure understood as traditional normative expectations conditions the association between career stress and identity exploration. The core constructs, theoretical foundations, and hypothesized relationships are summarized in **Figure 1**, which provides an overview of the study’s analytical framework. By integrating structural uncertainty, developmental processes, and contextual normative constraints within a single analytical framework, this approach seeks to address the limitations of earlier work by situating identity-related adaptation and well-being within a coherent, multilevel perspective.

## 2 Literature review

### 2.1 Future anxiety as a multilevel psychological structure with sociocultural roots

Future anxiety has been widely conceptualized as a central component of youth anxiety, characterized by a persistent concern with uncertainty and diminished perceived control over future life conditions rather than transient or short-lived emotional reactions. Evidence from large-scale societal disruptions further suggests that such future-oriented anxiety is systematically amplified in contexts marked by unpredictability and disrupted life planning, as observed during the COVID-19 pandemic (13). Converging evidence from neuroimaging research suggests that when individuals confront uncertainty about the future, neural systems associated with error monitoring—particularly within the anterior cingulate cortex and prefrontal regions—exhibit heightened activation, reflecting a sustained state of anticipatory vigilance rather than momentary fear (14).At the psychological level, future anxiety has been shown to co-occur with weakened self-concept coherence. da Silva Domingues et al. (15), for example, found that anxiety among university students was mediated by self-consistency and self-esteem, indicating that fragmented self-perceptions are closely linked to diminished agency and reduced perceived control over future outcomes. From a developmental perspective, this vulnerability is especially pronounced during adolescence and emerging adulthood, a period marked by delayed role transitions and prolonged dependence on educational, familial, and institutional structures (16).Importantly, cultural psychology highlights that future anxiety is not solely an individual cognitive phenomenon but is embedded within broader sociocultural contexts that shape meaning, identity, and agency. Usborne and de la Sablonnière (17) demonstrated that clarity of cultural identity contributes to stronger self-concept clarity and better mental health, whereas periods of cultural transition may blur identity boundaries and weaken agency. Similarly, Umaña-Taylor (18) showed that cultural identity socialization fosters resilience, enabling young people to cope more effectively with uncertainty. Taken together, these lines of evidence suggest that future anxiety is best understood as a multilevel psychological structure with clear sociocultural roots, spanning neural vigilance, self-related psychological processes, developmental timing, and culturally grounded systems of meaning that jointly shape how young people interpret uncertainty and regulate emotional experience.

### 2.2 Career stress, social comparison, and the transmission of anxiety

Career stress represents a critical context in which future anxiety becomes salient and intensified, as concerns about employability, competence, and life-course timing converge during the transition to adulthood. Early research on career decision-making difficulties highlighted how vague self-concepts, insufficient information, and conflicting expectations from families or institutions generate strong feelings of uncertainty and psychological distress (19). More recently, large-scale societal changes—particularly automation and technological transformation—have further destabilized traditional career pathways, increasing perceived employment uncertainty among young people (20).In contemporary settings, persistent social comparison further amplifies these pressures, particularly within digitally mediated environments where information about peers’ achievements and trajectories is continuously visible. A meta-analysis by Vogel et al. (21) showed that frequent engagement in social comparison is negatively associated with self-esteem, and that declines in self-esteem lead young people to evaluate their abilities more harshly and to feel less capable of meeting future career demands, thereby intensifying anxiety. Consistent with this pattern, population-level evidence indicates sustained increases in psychological distress among young people, suggesting that repeated social comparison and perceived inadequacy have become structurally embedded features of contemporary youth experience (22).Psychological resources play an important moderating role in this process. Monteiro et al. (23) found that career adaptability supports emotional regulation, future planning, and smoother school-to-work transitions, allowing individuals to maintain psychological stability under uncertain conditions. Cortellazzo et al. (24) further reported that individuals with a protean career orientation, characterized by autonomy and self-directed learning, are less reliant on external validation and therefore experience lower levels of anxiety associated with social comparison. At the structural level, technological transformation continues to reshape the sources and intensity of career stress. Bankins et al. (25) noted that advances in artificial intelligence are disrupting traditional linear career trajectories, requiring young people to continuously update skills and renegotiate professional identity. In addition to technological change, institutional dynamics may further magnify competitive pressure. In the Chinese context, Lin et al. (26) observed that credential inflation has intensified competition and led many university students to perceive a devaluation of academic qualifications, producing heightened anxiety even among highly educated groups. Overall, career-related stress and social comparison function not merely as external pressures but as internalized psychological processes through which self-evaluation and perceived control are continually adjusted, thereby sustaining anxiety within contemporary youth experience.

### 2.3 Identity exploration, integration, and psychological regulation

Identity exploration constitutes a central developmental task in youth and functions as a key psychological regulatory process in contexts of uncertainty. Drawing on the expanded four-dimensional model of identity formation, Luyckx et al. (27) described identity development as involving exploration, commitment, reconsideration, and reflection, while emphasizing that not all forms of exploration are adaptive. Ruminative exploration, in particular, has been associated with elevated anxiety, emotional exhaustion, and reduced well-being. Consistent with this view, Crocetti (28) emphasized that identity development unfolds through dynamic cycles of exploration and reconsideration, such that difficulties in integrating emerging commitments may intensify psychological distress rather than alleviate it. Cross-cultural research further indicates that pathways of identity exploration are deeply shaped by social norms and cultural expectations. Schwartz et al. (29) showed that although identity formation is a universal developmental task, the balance between autonomy and belonging varies substantially across cultural contexts. Building on this perspective, Syed and McLean (30) proposed the concept of identity integration, emphasizing narrative reflection and the synthesis of lived experience as foundations of psychological coherence. Longitudinal evidence supports this view: Nelson (31) found that increases in identity integration across emerging adulthood reliably predict improvements in well-being and mental health, suggesting that identity stability functions not as a fixed endpoint but as an ongoing form of psychological regulation. From a career development perspective, Savickas and Porfeli (32) similarly argued that adaptive identity construction relies on psychosocial resources that enable individuals to integrate changing roles, values, and future orientations under conditions of uncertainty. Research on cultural and ethnic identity development further underscores the protective role of social context. Pidduck et al. (33) demonstrated that cultural intelligence promotes openness and reflective processing during intercultural experiences, thereby supporting emotional balance, while Umaña-Taylor (18) similarly showed that positive cultural identity socialization enhances resilience among youth facing uncertainty. At the same time, identity exploration may be constrained or redirected by normative expectations embedded within family, community, and generational contexts. Homer (34) noted that under strong economic and cultural pressures, young people may internalize intergenerational expectations that limit exploratory space and require psychological compromise, shaping how identity-related stress is experienced and regulated. Taken together, these findings suggest that identity exploration contributes to psychological adaptation when supported by integration, resilience, and culturally grounded meaning systems rather than intensifying anxiety.

### 2.4 Toward an integrated chain model of youth anxiety

Synthesizing the literature reviewed above, youth anxiety can be understood as a psychological structure emerging from the interrelated influences of future anxiety, career-related stress, identity exploration, and sociocultural context. Drawing on the career shock framework, Akkermans et al. (35) suggested that sudden social disruptions undermine perceived control over the future and intensify anxiety, while cultural-psychological research similarly emphasizes that youth anxiety reflects not only economic instability but also the erosion of shared values and identity anchors (36). At the individual level, self-directed career orientations and learning-based regulatory strategies have been shown to support agency and emotional balance during periods of transition (37, 38). At the structural level, Cheng et al. (39) demonstrated that misalignment among skills, academic performance, and motivational factors reduces employment quality and psychological adaptation. Guided by this body of theoretical and empirical work, the present study proposes a chain-structured model linking future insecurity, career stress, and identity exploration, while examining the moderating role of sociocultural norms. Rather than implying causal mechanisms, this framework provides a model-based representation of how multiple psychological and contextual factors co-occur and interact under conditions of social uncertainty, shaping youth anxiety, well-being, and psychological adaptation.

## 3 Methods

### 3.1 Research design

A quantitative, cross-sectional research design was adopted to examine the associations among future anxiety, career stress, identity exploration, rural normative pressure, and well-being in a sample of Chinese university students. The study was guided by an integrative perspective that combined psychological and sociocultural approaches, with the aim of situating individual psychological experiences within broader contexts of uncertainty, transition, and social expectation. Drawing on cultural psychology, social comparison theory, and related perspectives on youth development under uncertainty, the study specified a conceptual framework in which future anxiety was modeled as an antecedent variable, career stress and identity exploration as intermediate psychological processes, rural normative pressure as a contextual moderator, and well-being as the primary outcome variable. More specifically, the model proposed that future anxiety would be associated with well-being both directly and indirectly through career stress and identity exploration, while rural normative pressure would moderate the association between career stress and identity exploration. The design was intended to test theoretically informed patterns of association within a structural framework rather than to establish temporal ordering or causal relationships.

### 3.2 Participants and procedure

Participants were undergraduate students recruited from several universities across mainland China. The study focused on young adults enrolled in higher education because this population occupies a developmental stage marked by increasing exposure to academic demands, career-related uncertainty, and shifting social expectations. A combination of stratified and convenience sampling was used across multiple institutions to enhance variability in key demographic characteristics, including gender, academic year, and urban–rural background, and recruitment was conducted through course announcements and online academic platforms commonly used by university students. Data were collected using an anonymous self-report questionnaire. Participation was entirely voluntary, and informed consent was obtained from all respondents before survey completion. Participants were informed of the general purpose of the study, assured that their responses would remain confidential, and told that they could withdraw from the survey at any time without penalty. After data collection, the responses were screened for completeness and quality, and cases with substantial missing data, invariant response patterns, or evident inattentive responding were excluded prior to analysis. After this screening process, 260 valid questionnaires were retained for the final analyses. Before data collection, the target sample size was determined with reference to commonly used recommendations for structural equation modeling. Given the complexity of the proposed model, which included multiple latent variables, indirect pathways, and a moderated association, a minimum sample size of approximately 200 was considered necessary to support stable parameter estimation and adequate statistical power. To account for potential case loss during data screening, the initial recruitment targeted a larger response pool. The final sample of 260 participants exceeded this threshold and was therefore considered sufficient for the planned analyses. The final sample comprised 50.4% male and 49.6% female participants, with ages ranging from 18 to 23 years (M = 20.43, SD = 1.73). In terms of residential background, 55.0% of participants reported urban origins and 45.0% reported rural origins, indicating a relatively balanced distribution. Socioeconomic status (SES) was assessed through self-reported family income and showed moderate variability within the sample (M = 3.21, SD = 0.46). To account for potential demographic influences on the focal psychological variables, gender, age, family SES, and urban–rural background were included as control variables in the analytical models. All measures were self-reported, and internal consistency checks, including reverse-coded items where applicable, were used to enhance data quality.

### 3.3 Measurement framework

This study developed a comprehensive measurement framework grounded in established psychological and sociocultural theories to examine the interrelationships among future anxiety, career stress, identity exploration, rural normative pressure, and well-being, while also incorporating key demographic variables as controls. The selection and design of the measurement indicators were guided by two core principles, namely theoretical coherence and empirical testability. Accordingly, each construct was operationalized with reference to established and widely used scales in the fields of youth development, career psychology, identity formation, and subjective well-being. The psychological constructs were organized hierarchically, with latent variables representing broader psychological processes and observed indicators capturing their measurable dimensions across emotional experience, cognitive appraisal, behavioral orientation, and sociocultural context. Specifically, future anxiety was measured with reference to the Future Anxiety Scale proposed by Zaleski (40), which conceptualizes future anxiety as a relatively enduring cognitive-emotional orientation marked by anticipated uncertainty, perceived lack of control, and pessimistic expectations about future life conditions. career stress was assessed using indicators adapted from established research on career stress and career decision-making difficulties, particularly the framework developed by Gati et al. (19), with an emphasis on stress arising from career choice, feelings of inadequacy, and comparison with others during the career preparation process. Identity exploration was measured using indicators derived from integrative models of identity development that conceptualize exploration as a central regulatory process in emerging adulthood (41), capturing self-reflection, directional exploration, value integration, and the active reconsideration of life goals. Rural normative pressure was operationalized as a context-specific sociocultural construct reflecting perceived traditional expectations in rural settings, including family obligations, kinship responsibility, collective reputation, and sensitivity to social evaluation. In the present study, this construct was theorized primarily as a contextual moderator shaping the strength of the association between career stress and identity exploration, rather than as a core direct outcome predictor. Well-being, treated as the primary outcome variable, was measured with reference to established subjective well-being research, particularly the tradition represented by Diener et al. (42), and was used to capture respondents’ overall level of positive psychological functioning and life evaluation under conditions of uncertainty. All measurement instruments were drawn from widely used international scales and were translated and adapted for the Chinese cultural context through standard translation and back-translation procedures. This process was supplemented by pilot testing to ensure semantic clarity, cultural appropriateness, and conceptual alignment between the original and localized versions of the instruments. As summarized in **Appendix A1**, all latent constructs were operationalized using multiple observed indicators. Internal consistency reliability and convergent validity were evaluated using Cronbach’s alpha, composite reliability (CR), and average variance extracted (AVE), with the corresponding results reported in **Table 1**. In addition, discriminant validity was assessed using both the Fornell–Larcker criterion and the heterotrait–monotrait ratio, as reported in **Table 2** and **3**. Overall, the measurement results indicated acceptable to good reliability, satisfactory convergent validity, and adequate discriminant validity, thereby providing a sound empirical foundation for the subsequent structural equation modeling analyses.

**Table 1:** Reliability and convergent validity of measurement constructs.

Variable	Cronbach’s α	CR	AVE
Future Anxiety (A)	0.924	0.952	0.868
career stress (B)	0.842	0.905	0.76
Identity Exploration (C)	0.823	0.894	0.739
Rural Normative Pressure (D)	0.832	0.923	0.856
Well-being (E)	0.836	0.924	0.859

**Table 2:** Discriminant validity: Fornell–Larcker criterion.

Variable	Future anxiety	Career stress	Identity exploration	Rural normative pressure	Well-being
Future Anxiety	0.932				
career stress	0.637	0.872			
Identity Exploration	0.538	0.542	0.86		
Rural Normative Pressure	0.322	0.301	0.314	0.925	
Well-being	-0.454	-0.422	-0.125	-0.202	0.927

Diagonal elements represent the square roots of the average variance extracted (AVE); off-diagonal elements represent inter-construct correlations. Discriminant validity is supported when each diagonal value exceeds the corresponding off-diagonal correlations in the same row and column.

**Table 3:** Discriminant validity: heterotrait–monotrait ratio (HTMT) matrix.

Variable	Future anxiety	Career stress	Identity exploration	Rural normative pressure	Well-being
Future Anxiety		0.722	0.617	0.367	0.516
career stress	0.722		0.649	0.36	0.502
Identity Exploration	0.617	0.649		0.379	0.152
Rural Normative Pressure	0.367	0.36	0.379		0.242
Well-being	0.516	0.502	0.152	0.242	

### 3.4 Analytical strategy

To examine the pattern of associations among future anxiety, career stress, identity exploration, rural normative pressure, and well-being, the analyses were conducted in a Python-based statistical environment. Data preprocessing and scale-score construction were performed using pandas and NumPy, correlational analyses and significance testing were conducted using SciPy, regression-based analyses were performed using statsmodels, and measurement and structural models were estimated using semopy. Spreadsheet outputs for the final results tables were generated using openpyxl. This analytical framework was adopted because it allowed the study to combine psychometric evaluation, regression-based moderation analysis, and structural equation modeling within a single reproducible workflow. In the present study, the modeling strategy was used to assess the structural coherence of the proposed framework and the magnitude of model-based associations among the study variables, rather than to imply causal mechanisms. The analytical procedure proceeded in several stages. First, internal consistency reliability and convergent validity were evaluated using Cronbach’s alpha, composite reliability (CR), and average variance extracted (AVE). Second, discriminant validity was assessed using both the Fornell–Larcker criterion and the heterotrait–monotrait ratio (HTMT). Third, a confirmatory factor analysis (CFA) was conducted to evaluate the hypothesized five-factor measurement model, and alternative four-factor, three-factor, and one-factor models were compared to assess the distinctiveness of the latent constructs; the standardized factor loadings of the final five-factor model are reported in **Appendix A2**. Fourth, while the overall framework is conceptualized within a structural equation modeling tradition, the direct path coefficients reported in **Table 4** and the moderation and simple slope estimates reported in **Table 5** were obtained through a series of OLS regression equations using standardized variables, implemented via statsmodels. This approach was adopted to enable flexible incorporation of the interaction term for moderation testing within the same analytical pipeline. Measurement model evaluation and model fit comparisons reported in **Tables 1**–**7** were conducted using full SEM estimation via semopy. Indirect effects were then estimated using bootstrap resampling with 5,000 iterations to assess the specific indirect pathways linking future anxiety to well-being through career stress and identity exploration. Fifth, moderation analysis was conducted to examine whether rural normative pressure altered the strength of the association between career stress and identity exploration, and simple slope analysis was used to clarify the interaction pattern. Finally, supplementary group-comparison analyses were conducted across key demographic subgroups to examine the robustness of the proposed model. Model fit was evaluated using multiple commonly reported indices, including χ²/df, CFI, TLI, RMSEA, and SRMR. In line with commonly used guidelines, χ²/df values below 3.00 were taken to indicate acceptable fit, CFI and TLI values above 0.90 acceptable fit and above 0.95 good fit, RMSEA values below 0.08 acceptable fit and below 0.06 good fit, and SRMR values below 0.08 acceptable fit. Readers should note that the OLS-based path estimates and the SEM-based fit indices reflect complementary but technically distinct estimation procedures, and results should be interpreted accordingly.

**Table 4:** Structural path coefficients of the chain mediation model.

Path	β	SE	t	p
Future Anxiety → career stress	0.637	0.048	13.259	<0.001
Future Anxiety → Identity Exploration	0.346	0.065	5.317	<0.001
career stress → Identity Exploration	0.316	0.065	4.873	<0.001
Rural Normative Pressure moderates career stress → Identity Exploration	0.112	0.05	2.235	0.026
Future Anxiety → Well-being	-0.383	0.073	-5.265	<0.001
career stress → Well-being	-0.3	0.073	-4.135	<0.001
Identity Exploration → Well-being	0.265	0.067	3.968	<0.001
Rural Normative Pressure → Well-being	-0.071	0.057	-1.242	0.216

Standardized path coefficients (β) are reported. The moderation row reflects the interaction term (Career Stress × Rural Normative Pressure) estimated within a model regressing Identity Exploration on Future Anxiety, Career Stress, and the Career Stress × Rural Normative Pressure interaction term; the main effect of Rural Normative Pressure was not included in this equation. All other paths were estimated in separate OLS regression equations with standardized variables. SE, standard error.

**Table 5:** Moderation and simple slope analysis of rural–normative pressure.

Effect	β	SE	t	p
Interaction term (B × D → C)	0.105	0.051	2.064	0.04
Simple slope (High +1 SD)	0.405	0.078	5.2	<0.001
Simple slope (Mean)	0.299	0.065	4.61	<0.001
Simple slope (Low −1 SD)	0.194	0.087	2.231	0.027

Standardized coefficients (β) are reported. The interaction term and simple slopes were estimated from a model regressing Identity Exploration on Future Anxiety (A), career stress (B), Rural Normative Pressure (D), and the B × D interaction term, with all predictors standardized prior to entry. Simple slopes represent the association between career stress and Identity Exploration at high (+1 SD), mean, and low (−1 SD) levels of Rural Normative Pressure. SE, standard error.

**Table 6:** Means, standard deviations, and correlations among key variables (N = 260).

Variable	Mean	SD	1	2	3	4	5
Future Anxiety	3.34	1.11	1.000				
career stress	2.26	0.89	0.637***	1.000			
Identity Exploration	2.13	0.88	0.538***	0.542***	1.000		
Rural Normative Pressure	2.78	1.03	0.322***	0.301***	0.314***	1.000	
Well-being	2.91	1.02	-0.454***	-0.422***	-0.125*	-0.202**	1.000

The symbol *** indicates p < 0.001.

**Table 7:** Comparison of fit indices for the measurement and structural models.

Model	χ²	df	χ²/df	CFI	TLI	RMSEA	SRMR
Five-factor measurement model	55.758	55	1.014	1	0.999	0.007	0.026
Four-factor model (A and B combined)	206.892	59	3.507	0.923	0.898	0.098	0.061
Three-factor model	378.58	62	6.106	0.835	0.793	0.14	0.093
One-factor model	678.411	65	10.437	0.681	0.617	0.191	0.128
Final structural model	62.703	57	1.1	0.997	0.996	0.02	0.04

Lower χ²/df, RMSEA, and SRMR values and higher CFI and TLI values indicate better model fit. The five-factor measurement model and the final structural model showed substantially better fit than the alternative models, supporting the distinctiveness of the proposed constructs and the adequacy of the hypothesized structural framework.

## 4 Results

### 4.1 Reliability, validity, and preliminary descriptive statistics

**Table 1** shows that all core constructs demonstrated good internal consistency and convergent validity. Cronbach’s alpha values ranged from 0.823 to 0.924, composite reliability values ranged from 0.894 to 0.952, and average variance extracted values ranged from 0.739 to 0.868, all meeting or exceeding commonly accepted standards. These findings indicate that the indicators were consistently related to their intended latent variables and that each construct accounted for a substantial proportion of variance in its indicators. Taken together, the results provide a sound measurement basis for the subsequent correlational, structural, mediation, and moderation analyses. At the same time, these results primarily support reliability and convergent validity; discriminant validity must be further evaluated using the Fornell–Larcker and HTMT results reported in the following tables.

**Table 6** reports the descriptive statistics and zero-order correlations among the key variables. Future Anxiety was positively correlated with career stress (r = 0.637, p <.001) and Identity Exploration (r = 0.538, p <.001). career stress was also positively correlated with Identity Exploration (r = 0.542, p <.001). Well-being was negatively correlated with Future Anxiety (r = -0.454, p <.001), career stress (r = -0.422, p <.001), Identity Exploration (r = -0.125, p <.05), and Rural Normative Pressure (r = -0.202, p <.01). Overall, the correlations showed that the focal variables were significantly associated in the expected directions.

**Table 2** evaluates discriminant validity using the Fornell–Larcker criterion. The square roots of the AVE values, shown on the diagonal, ranged from 0.860 to 0.932 and were all greater than the corresponding inter-construct correlations. These results support discriminant validity for Future Anxiety, career stress, Identity Exploration, Rural Normative Pressure, and Well-being. Although the correlation between Future Anxiety and career stress was relatively high (r = 0.637), the diagonal values for both constructs remained larger than the corresponding off-diagonal correlation.

**Table 3** further examines discriminant validity using the Heterotrait–Monotrait ratio. All HTMT values ranged from 0.152 to 0.722, remaining well below the commonly recommended cutoffs of 0.85 or 0.90. This pattern indicates that the latent constructs were empirically distinguishable and did not exhibit problematic overlap. In particular, although Future Anxiety, career stress, and Identity Exploration were moderately related, their HTMT values remained within an acceptable range, suggesting that they reflected related but nonredundant dimensions of the proposed framework. Together with the Fornell–Larcker evidence reported in **Table 2**, the HTMT findings provide strong support for the discriminant validity of the measurement model.

### 4.2 Measurement and structural model fit

**Table 7** compares the fit indices of the measurement, competing, and structural models. The hypothesized five-factor measurement model showed excellent fit to the data (χ²/df = 1.014, CFI = 1.000, TLI = 0.999, RMSEA = 0.007, SRMR = 0.026) and fit the data better than the four-factor, three-factor, and one-factor alternatives. The final structural model also showed good fit (χ²/df = 1.100, CFI = 0.997, TLI = 0.996, RMSEA = 0.020, SRMR = 0.040). Overall, these results support the adequacy of the measurement model and the proposed structural model.

### 4.3 Structural model testing: the chain mediation mechanism

**Figure 2:**
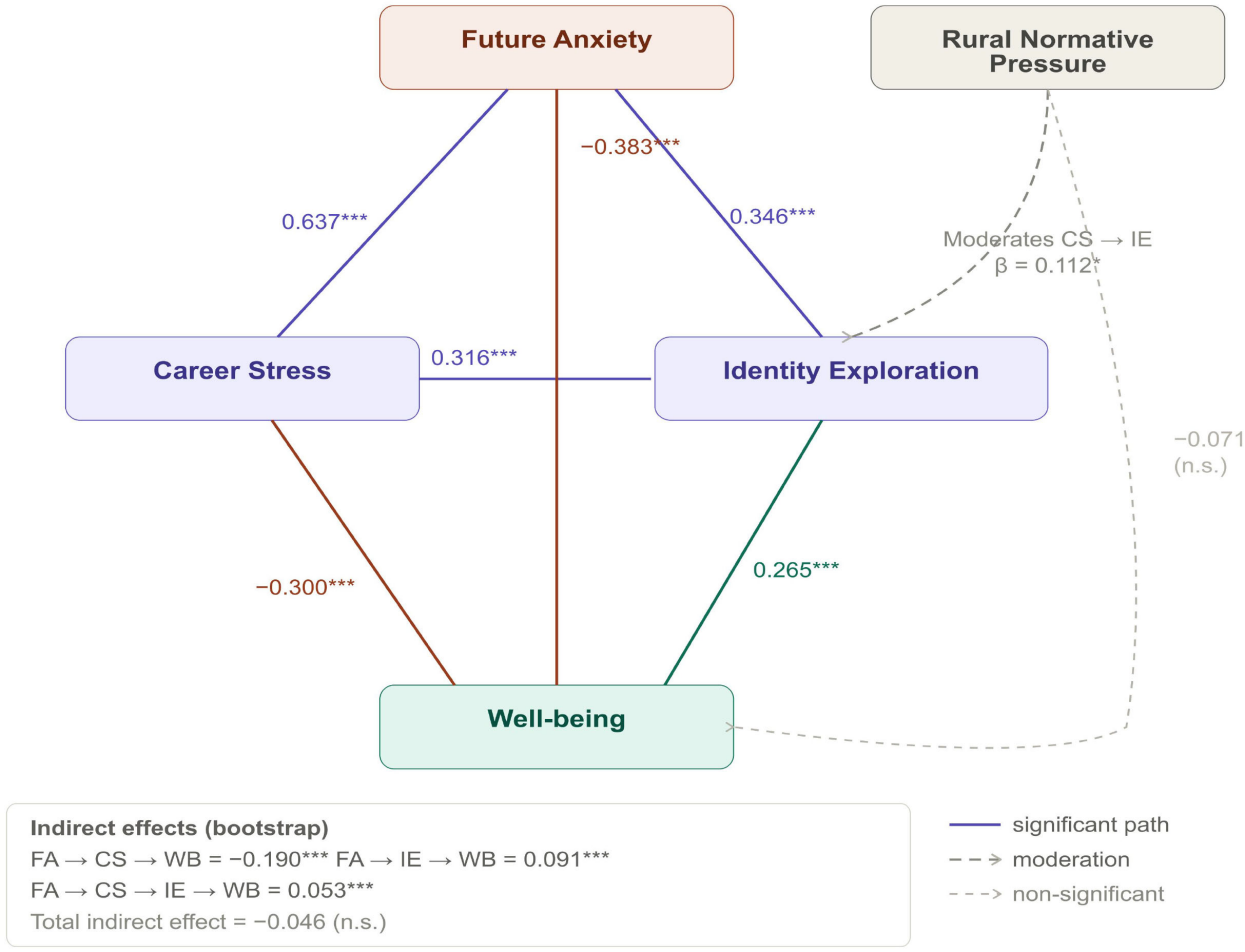
Empirical structural model with direct and indirect effects. The symbol *** indicates p < 0.001.

**Table 4** and **Figure 2** present the standardized path coefficients of the chain mediation model. Future anxiety positively predicted career stress (β = 0.637, p <.001) and identity exploration (β = 0.346, p <.001), and career stress also positively predicted identity exploration (β = 0.316, p <.001). For well-being, future anxiety (β = -0.383, p <.001) and career stress (β = -0.300, p <.001) were both negative predictors, whereas identity exploration was a positive predictor in the multivariate model (β = 0.265, p <.001). The direct effect of rural normative pressure on well-being was negative but not significant (β = -0.071, p = .216).

**Table 8** reports the bootstrap estimates of the indirect effects, which are also summarized in **Figure 2**. The indirect effect from Future Anxiety to Well-being through career stress was significant (β = -0.190, 95% CI [-0.286, -0.096], p <.001). The indirect effect through Identity Exploration was also significant (β = 0.091, 95% CI [0.043, 0.149], p <.001). In addition, the sequential indirect effect through career stress and Identity Exploration was significant (β = 0.053, 95% CI [0.023, 0.091], p <.001). However, the total indirect effect of Future Anxiety on Well-being was not significant (β = -0.046, 95% CI [-0.148, 0.057], p = .373).

**Table 8:** Indirect effects (bootstrap, 5000 samples).

Effect	β	95% CI	p
A → B → E	-0.19	[-0.286, -0.096]	<0.001
A → C → E	0.091	[0.043, 0.149]	<0.001
A → B → C → E	0.053	[0.023, 0.091]	<0.001
Total indirect effect of A on E	-0.046	[-0.148, 0.057]	0.373

### 4.4 Moderating effect of rural–normative pressure

**Figure 3:**
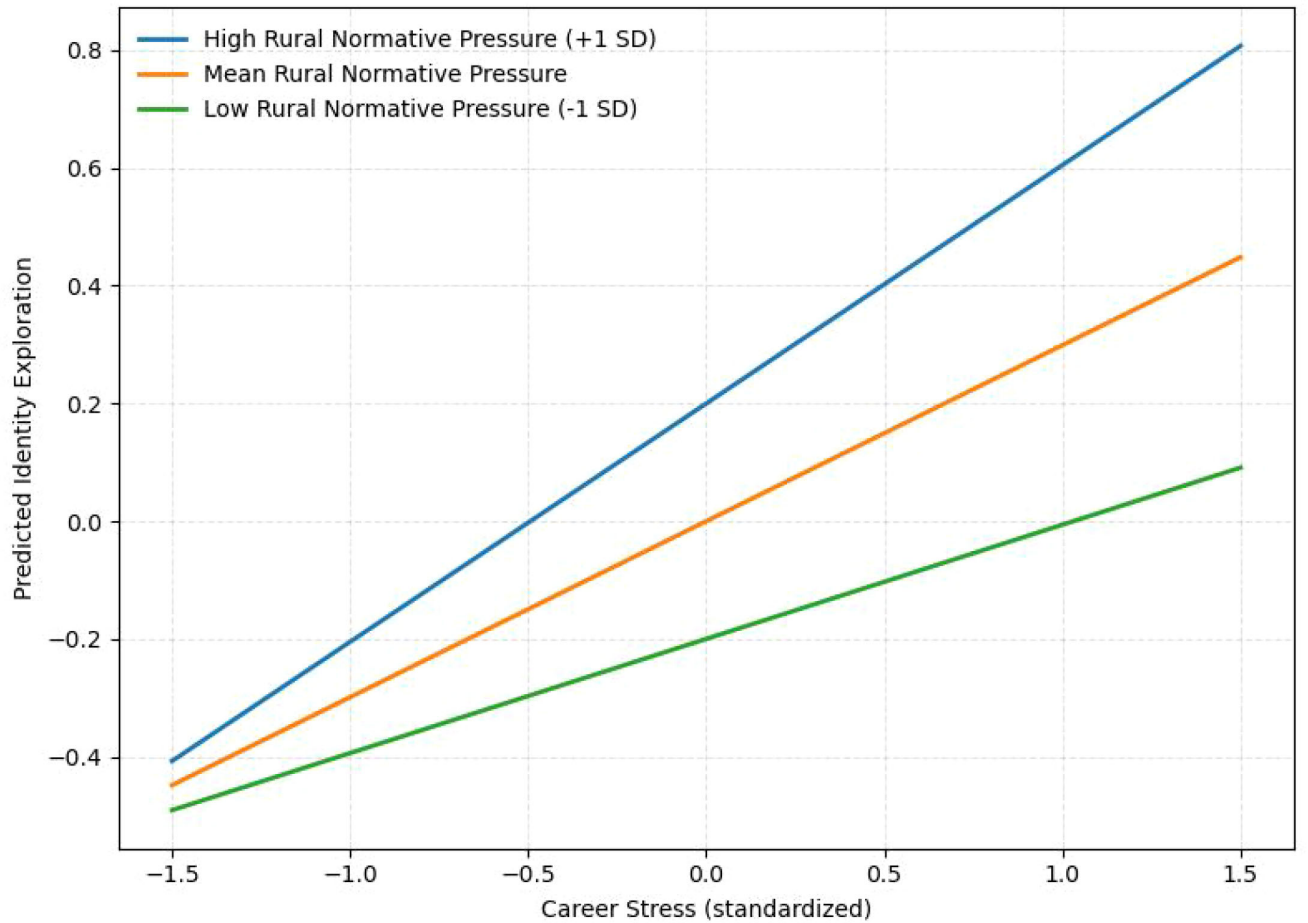
Simple slope plot illustrating the moderating effect of rural–normative pressure on the relationship between career stress and identity exploration.

**Table 5** tests the moderating role of Rural Normative Pressure in the association between career stress and Identity Exploration. The interaction term was significant (β = 0.105, p = .040). The simple slope analysis showed that the positive association between career stress and Identity Exploration was strongest at high levels of Rural Normative Pressure (β = 0.405, p <.001), remained significant at the mean level (β = 0.299, p <.001), and was weaker but still significant at low levels (β = 0.194, p = .027). **Figure 3** illustrates this pattern.

### 4.5 Multi group and control variable analysis

**Table 9** presents the multi-group robustness checks across gender, urban-rural background, and socioeconomic status. The chi-square difference tests were non-significant for all three comparisons: Gender (Δχ² = 0.846, Δdf = 5, p = .518), Urban-Rural (Δχ² = 0.281, Δdf = 5, p = .924), and SES (Δχ² = 1.019, Δdf = 5, p = .407). The corresponding ΔCFI values were 0.000, 0.006, and 0.011, respectively. Overall, these results indicate that the model showed generally comparable structural patterns across the examined demographic subgroups.

**Table 9:** Multi-group invariance tests as robustness checks.

Group comparison	Δχ²	Δdf	p	ΔCFI
Gender (M/F)	0.846	5	0.518	0
Urban–Rural	0.281	5	0.924	0.006
SES (High/Low)	1.019	5	0.407	0.011

## 5 Discussion

### 5.1 Integrating future anxiety, career stress, and identity exploration under uncertainty

The integration of future anxiety, career stress, and identity exploration in the present study points to a broader developmental reality in which young people confront prolonged uncertainty not through isolated psychological reactions, but through interrelated processes of pressure, reflection, and self-reorganization. Empirically, future anxiety was strongly associated with both career stress and identity exploration at the correlational level (r = 0.637 and r = 0.538, respectively; **Table 6**), and these associations remained significant in the structural model, where future anxiety positively predicted career stress (β = 0.637) and identity exploration (β = 0.346), while career stress also positively predicted identity exploration (β = 0.316; **Table 4**). Taken together, these findings suggest that uncertainty about the future, pressure surrounding career preparation, and active reflection on one’s identity are not separate experiences, but intertwined features of adaptation in contemporary youth. Rather than responding to a single temporary stressor, many young people are navigating a social world in which future planning has become persistent, consequential, and increasingly difficult. Educational expansion, intensified competition, and unstable opportunity structures require them to make meaningful decisions about who they are and where they are going, often in the absence of clear or reliable reference points. Within such conditions, identity exploration emerges alongside stress not as a secondary or optional developmental task, but as a psychologically meaningful response to ongoing demands for self-definition.

A particularly important aspect of the findings is that identity exploration showed a weak negative zero-order correlation with well-being (r = -0.125; **Table 6**), yet a positive association in the multivariate model (β = 0.265; **Table 4**). This contrast suggests that the role of identity exploration is more complex than a simple risk-versus-benefit distinction would imply. At the bivariate level, identity exploration may appear burdensome because it is entangled with uncertainty and stress; however, once the shared variance associated with future anxiety and career stress is taken into account, its more constructive developmental function becomes more visible. In this sense, identity exploration may reflect not only distress, but also an effort to reorganize goals, values, and self-understanding under unstable conditions. Such an interpretation is consistent with identity integration perspectives, which emphasize that reflective engagement with one’s future can support psychological coherence when it is not overwhelmed by co-occurring psychological burdens. Overall, the present findings suggest that anxiety, pressure, and identity work are deeply intertwined in contemporary youth experience, and that adaptation under uncertainty involves not only strain, but also attempts at meaning-making and developmental reorientation.

### 5.2 Rural normative pressure as a contextual amplifier of identity-related adaptation

The moderating role of rural normative pressure highlights how sociocultural context conditions, rather than determines, the ways in which young people experience and respond to career-related strain. Empirically, rural normative pressure showed a significant interaction with career stress in predicting identity exploration (β = 0.105, p = .040; **Table 5**), and the simple slope analysis further indicated that the positive association between career stress and identity exploration became progressively stronger as rural normative pressure increased, being strongest at high levels of rural normative pressure (β = 0.405), remaining significant at the mean level (β = 0.299), and becoming weaker, though still significant, at low levels (β = 0.194). These findings suggest that sociocultural expectations do not alter the overall direction of the association between stress and identity exploration, but they do shape the intensity with which the two are connected. In this sense, rural normative pressure appears to function less as a direct psychological determinant than as a contextual amplifier that strengthens the extent to which career-related pressure becomes tied to questions of self-definition. This interpretation is also consistent with the non-significant direct effect of rural normative pressure on well-being (β = -0.071, p = .216; **Table 4**), which suggests that its role in the present model is better understood as contextual than as directly outcome-generating. In substantive terms, this pattern suggests that young people embedded in stronger normative environments are often confronted with more explicit expectations concerning responsibility, persistence, family obligation, and socially sanctioned life trajectories. Under such conditions, career-related stress may become more consequential not simply because pressure increases, but because pressure carries stronger implications for who one is expected to become. Identity-related reflection therefore becomes more likely when social evaluation is more salient and when future choices are more closely tied to moral, familial, or reputational expectations. From this perspective, rural normative pressure does not directly produce exploration; rather, it intensifies the conditions under which stress is translated into self-reflection. The broader robustness results are consistent with this interpretation. The multi-group analyses showed generally comparable structural patterns across gender, urban–rural background, and socioeconomic status (**Table 9**), suggesting that the overall framework was not confined to a single subgroup, although the slight SES-related fluctuation also indicates that social position and available resources may still influence how strongly these processes are experienced in everyday life. Taken together, the findings suggest that sociocultural norms function less as fixed cultural determinants than as contextual conditions that intensify the lived connection between pressure and identity work while leaving the broader structural pattern intact.

### 5.3 Opposing indirect pathways from future anxiety to well-being

One particularly important finding is that the indirect association between future anxiety and well-being was not uniform, but operated through multiple pathways in opposite directions. Empirically, future anxiety was directly associated with lower well-being (β = -0.383, p <.001), while also positively predicting career stress (β = 0.637, p <.001) and identity exploration (β = 0.346, p <.001; **Table 4**). career stress, in turn, was negatively associated with well-being (β = -0.300, p <.001) but positively associated with identity exploration (β = 0.316, p <.001), whereas identity exploration showed a positive association with well-being in the multivariate model (β = 0.265, p <.001). This overall structure was mirrored in the indirect effects reported in **Table 8**: the path from future anxiety to well-being through career stress was significant and negative (β = -0.190, 95% CI [-0.286, -0.096], p <.001), whereas the path through identity exploration was significant and positive (β = 0.091, 95% CI [0.043, 0.149], p <.001); in addition, the sequential indirect effect through career stress and identity exploration was also significant (β = 0.053, 95% CI [0.023, 0.091], p <.001). By contrast, the total indirect effect was not significant (β = -0.046, 95% CI [-0.148, 0.057], p = .373), a pattern consistent with inconsistent mediation, in which specific indirect pathways remain meaningful even though their aggregate effect is attenuated because they operate in opposing directions. Accordingly, the non-significance of the total indirect effect should not be interpreted as evidence that mediation is absent, but rather as an indication that the underlying mechanisms are competing rather than convergent. Substantively, this pattern suggests that future anxiety should not be understood solely as a psychological risk factor. On the one hand, it activates a depleting pathway through career stress that undermines well-being by intensifying pressure, insecurity, and perceived inadequacy. On the other hand, it is also linked to a more developmental pathway through identity exploration, in which uncertainty appears to stimulate reflection on values, direction, and self-definition, and this pathway becomes positively associated with well-being once the overlapping burden of anxiety and stress is taken into account. In this sense, the relationship between future anxiety and well-being is more complex than a simple deficit model would suggest, because the same uncertainty that generates strain may also prompt efforts at meaning-making and developmental reorientation. This dual-process structure has important practical implications: interventions aimed at supporting youth well-being under uncertainty may need to address not only stress reduction, but also the reflective and identity-related processes through which young people attempt to reorganize their future under unstable conditions.

### 5.4 Theoretical contributions, practical implications, and limitations

By integrating future anxiety, career stress, identity exploration, and rural normative pressure within a single analytical framework, the present study offers a more contextualized account of young people’s well-being under conditions of uncertainty. Rather than portraying future anxiety as a purely negative psychological state, the findings suggest that its association with well-being is more complex, operating through both depleting and potentially developmental pathways. On the one hand, future anxiety was linked to lower well-being both directly and indirectly through career stress; on the other hand, it was also positively associated with identity exploration, which in turn showed a positive association with well-being in the multivariate model. In this sense, the study extends existing research by showing that uncertainty does not merely undermine adaptation, but may also stimulate reflective processes through which young people attempt to reorganize goals, clarify identity, and preserve psychological functioning. The moderating effect of rural normative pressure further strengthens this contextual perspective by indicating that the link between career stress and identity exploration becomes stronger when normative expectations are more salient, thereby underscoring that psychological adaptation unfolds not only through internal responses to uncertainty, but also through the sociocultural environments in which stress, self-definition, and future planning are embedded. Practically, these findings suggest that efforts to support young people’s well-being should not focus exclusively on stress reduction. Interventions may also benefit from fostering reflective capacity, identity-related meaning-making, and more adaptive ways of interpreting future uncertainty, while remaining attentive to the normative and social contexts within which these developmental processes take shape. At the same time, several limitations should be acknowledged. First, because the study was cross-sectional, the observed structural pattern should not be interpreted as establishing temporal order or causal direction. Although the final measurement and structural models showed good fit, model fit alone does not by itself confirm causality, and longitudinal or experimental research would be needed to clarify how anxiety, stress, exploration, and well-being influence one another over time. Second, all variables were assessed through self-report measures, which leaves open the possibility of shared method variance and common response tendencies. Although the poor fit of the one-factor model provides some reassurance that the findings do not simply reflect a single undifferentiated factor, future studies could strengthen the robustness of the evidence by incorporating multi-source data, behavioral indicators, or time-lagged designs. Third, the sample was drawn from Chinese undergraduate students within a specific sociocultural and educational context, which may limit the generalizability of the findings to other age groups, nonstudent populations, or different cultural settings. Comparative and cross-cultural studies would therefore be useful for examining the broader applicability of the proposed framework. Fourth, the operationalization of rural normative pressure relied on only two indicators—family and kinship obligations, and collective reputation and social evaluation—which represents a relatively narrow measurement base for a construct with broader sociocultural dimensions. Although this two-indicator solution demonstrated acceptable reliability, it provided limited coverage of the construct’s full breadth. Important facets of rural normative context, including intergenerational obligation, marriage and fertility pressure, and community-level surveillance norms, were not captured. Future research should therefore develop more comprehensive multi-item measures of rural normative pressure and examine their discriminant validity in relation to related constructs such as collectivism and filial piety. Finally, although the multi-group analyses suggested that the overall structural pattern was broadly comparable across gender, urban–rural background, and socioeconomic status, the slight SES-related fluctuation indicates that subgroup variation may still warrant closer attention in future research. More rigorous multi-group SEM procedures and more diverse samples would help clarify whether the present framework operates with the same strength across different social positions. Taken together, the findings are best understood as illuminating patterns of adaptation and well-being in everyday developmental contexts rather than establishing fixed causal mechanisms or clinical processes.

## 6 Conclusion

This study examined how young people adapt psychologically under conditions of uncertainty by integrating future anxiety, career stress, identity exploration, and rural normative pressure within a single framework centered on well-being. The findings showed that future anxiety was associated with higher career stress (β = 0.637, p <.001) and lower well-being (β = -0.383, p <.001), suggesting that uncertainty about the future has become an important source of psychological burden for young adults. At the same time, future anxiety was also positively associated with identity exploration (β = 0.346, p <.001), while career stress likewise positively predicted identity exploration (β = 0.316, p <.001). These results indicate that uncertainty and pressure do not merely exhaust young people, but may also push them toward greater reflection on self-definition, life direction, and future roles. Identity exploration, in turn, showed a positive net association with well-being in the multivariate model (β = 0.265, p <.001), suggesting that reflective efforts to reorganize values and clarify one’s sense of self may carry developmental benefits that become apparent once co-occurring anxiety and stress are statistically accounted for. The indirect effect results further supported this interpretation. Future anxiety was linked to lower well-being through career stress (β = -0.190, p <.001), but it was also linked to higher well-being through identity exploration (β = 0.091, p <.001), and the sequential indirect effect through career stress and identity exploration was likewise significant (β = 0.053, p <.001). Notably, the total indirect effect was not significant (β = -0.046, p = .373), a pattern consistent with inconsistent mediation (43), in which opposing specific pathways partially cancel one another at the aggregate level. This result does not negate the individual pathways; rather, it reflects their competing nature and underscores why specific indirect effects warrant independent examination when theoretically distinct mechanisms are expected to operate simultaneously. The moderating results further showed that rural normative pressure shaped how stress was translated into self-reflection: the interaction between career stress and rural normative pressure was significant (β = 0.105, p = .040), and the positive association between career stress and identity exploration became stronger at higher levels of rural normative pressure (high: β = 0.405; mean: β = 0.299; low: β = 0.194). This pattern suggests that sociocultural norms do not simply determine adaptation, but instead intensify the degree to which pressure becomes tied to questions of identity when social expectations are more salient.

Taken together, the study extends previous work by moving beyond a one-dimensional view of youth anxiety and showing that uncertainty may simultaneously activate both depleting and developmental processes. From a theoretical perspective, the findings connect stress, exploration, and well-being within a sociocultural framework, highlighting how psychological adaptation is shaped not only by internal responses to uncertainty but also by the normative environments in which young people interpret their future. In practical terms, the results suggest that educational and policy efforts should not focus only on reducing stress, but also on supporting reflective capacity, identity-related meaning-making, and adaptive coping under social pressure. At the same time, the study has several limitations. Its cross-sectional design prevents causal inference, all variables were assessed through self-report measures, and the sample was limited to Chinese undergraduate students, which may restrict broader generalizability. Future research could build on these findings by using longitudinal, cross-cultural, and multi-method designs to examine how the balance between pressure, exploration, and well-being develops across different social and cultural contexts.

## Data Availability

The raw data supporting the conclusions of this article will be made available by the authors, without undue reservation.

## Appendix A1

**Table:** Latent constructs and their measurement indicators.

Latent variable	Observed variable
A. Future Anxiety	A1. Uncertainty Anticipation
	A2. Perceived Lack of Control
	A3. Future Pessimism
B. career stress	B1. Career Choice Anxiety
	B2. Perceived Inadequacy
	B3. Social Comparison Stress
C. Identity Exploration	C1. Self-Reflection and Direction
	C2. Value Integration
	C3. Goal Commitment
D. Rural Normative Pressure (moderating variable)	D1. Family and Kinship Obligations
	D2. Collective Reputation and Social Evaluation
E. Well-being (outcome variable)	E1. Life Satisfaction
	E2. Positive Psychological Functioning
F. Demographics (control variables)	F1. Gender
	F2. Age
	F3. Socioeconomic Status
	F4. Urban–Rural Background

## Appendix A2

**Table:** Standardized factor loadings of the five-factor measurement model.

Latent variable	Item	Standardized loading
Future Anxiety	A1	0.881
	A2	0.900
	A3	0.906
career stress	B1	0.787
	B2	0.817
	B3	0.796
Identity Exploration	C1	0.777
	C2	0.835
	C3	0.727
Rural Normative Pressure	D1	0.929
	D2	0.768
Well-being	E1	0.855
	E2	0.841
